# Metagenomes of Lichens Solorina crocea and Peltigera canina

**DOI:** 10.1128/MRA.01000-21

**Published:** 2022-01-06

**Authors:** Maxim V. Vecherskii, David R. Khayrullin, Andrey M. Shadrin, Alexander V. Lisov, Anna G. Zavarzina, Alexey A. Zavarzin, Alexey A. Leontievsky

**Affiliations:** a Severtsov Institute of Ecology and Evolution, Russian Academy of Sciences, Moscow, Russia; b G. K. Skryabin Institute of Biochemistry and Physiology of Microorganisms, Pushchino Scientific Center for Biological Research of the Russian Academy of Sciences, Federal Research Center, Pushchino, Moscow Oblast, Russia; c Faculty of Soil Science, Moscow State University, Moscow, Russia; d N. I. Vavilov All-Russian Institute of Plant Genetic Resources, St. Petersburg, Russia; University of California, Riverside

## Abstract

Lichen genomes are usually considered genomes of separately cultured mycobiont and photobiont. Analysis of lichen metagenomes can give important information on specific lichen-associated microorganisms that can affect lichen metabolism. Here, we report a metagenome of peltigeralean lichens, containing cyanobacterial (Peltigera canina) and cyanobacterial/green algal (Solorina crocea) partners.

## ANNOUNCEMENT

Lichens are symbiotic associations of a fungus and a photobiont (a green alga and/or a cyanobacterium). Lichen genomes are usually considered genomes of separately cultured mycobiont and photobiont. Recent studies have shown that lichens contain prokaryotic and mycotic inhabitants that can significantly affect lichen metabolism (1). The specificity of lichen-associated microorganisms is debated (1). Here, we report a metagenome of two epigeic lichens of the order *Peltigerales*, containing a cyanobacterium (Peltigera canina) and a green alga/cyanobacterium (Solorina crocea) as photobionts. These lichens are producers of laccase and tyrosinase (2) with unusual properties (3) and potentially important roles in lichen metabolism and soil organic matter turnover (4).

A thallus of Peltigera canina was collected from soil in the southern taiga zone (Moscow region [56.220188N, 38.005571E]), and a Solorina crocea thallus was collected on the southern slope of the Khibiny Mountains (Murmansk region [67.59479N, 33.58666E]). The thalli were cleaned of visible contamination, washed with tap water, and dried in air for long-term storage. Thirty to 40 mg of dry thalli was ground using a mortar and pestle with 0.6 mL of homogenization buffer (5), followed by homogenization in a TissueLyser LT instrument (Qiagen) for 20 min at 50 Hz in 2-mL test tubes with a 7-mm steel ball. Total DNA was isolated using the cetyltrimethylammonium bromide (CTAB) method, followed by phenol-chloroform extraction and isopropanol precipitation (5). Libraries were prepared using the KAPA HyperPlus kit (Roche) as recommended by the manufacturer and were sequenced on an Illumina MiSeq platform (101-bp paired-end reads). Raw reads were checked with FastQC v0.11.9 (6). All check results were good, and no overrepresented sequences were discovered. Reads were assembled into contigs using MEGAHIT v1.2.9 (7) with default settings. The assembly results are shown in Table 1.

**TABLE 1:** Metagenomic characteristics of P. canina and S. crocea

Parameter	Finding for:	
	P. canina	S. crocea
GPS coordinates	56.220188N, 38.005571E	67.59479N, 33.58666E
Total no. of reads	36,044,600	44,270,896
No. of contigs assembled	103,792	172,170
GenBank accession no.	JAIOUS000000000	JAIQCQ000000000
Total assembly length (bp)	143,396,456	232,276,274
Minimum contig length (bp)	200	200
Maximum contig length (bp)	305,214	180,077
Avg contig length (bp)	1,381	1,349
*N*_50_ (bp)	8,580	4,228
BioProject accession no.	PRJNA756680	PRJNA756777

Assembled P. canina contigs were checked with BLASTn v2.11.0+ (8) for Peltigera canina internal transcribed spacer 1 (ITS1), 5.8S rRNA, and ITS2 genes (99.5% similarity to GenBank accession number MT644898.1) and the *Nostoc* sp. 16S rRNA gene (100% similarity to GenBank accession number DQ185249.1). Besides this, the Peltigera canina metagenome contains the *Colletotrichum* sp. 18S rRNA gene (99.5% similarity to GenBank accession number AJ301957.1). The 10 largest contigs (305 to 210 kb), i.e., k119_40300, k119_43765, k119_127377, k119_90562, k119_9057, k119_92597, k119_52320, k119_62742, k119_64239, and k119_28823, were annotated as parts of the *Nostoc* genome. The largest annotated mycobiont contigs, k119_19895 and k119_106695, were smaller, i.e., 53 kb and 38 kb, respectively. The rest of the symbionts were represented by shorter contigs.

Assembled S. crocea contigs were checked with BLASTn v2.11.0+ for the Solorina crocea 18S rRNA gene (100% similarity to GenBank accession number KJ766796.1), the *Coccomyxa solorinae* 5.8S rRNA gene (100% similarity to GenBank accession number MH753231.1), the *Coccomyxa* ribulose-1,5-bisphosphate carboxylase subunit (99.9% similarity to GenBank accession number JF502543.1), and the *Nostoc* sp. 16S rRNA gene (95% similarity to GenBank accession number KF359687.1). Besides this, the Solorina crocea metagenome contains the *Caulobacteraceae* bacterium 16S rRNA gene (96.7% similarity to GenBank accession number JQ402839.1), the *Trichoderma* sp. 18S rRNA gene (99.5% similarity to GenBank accession number AY489694), and the Bryidae moss 18S rRNA gene (100% similarity to GenBank accession numbers KC291527.1 and AF023709.1). The latter is a contaminant, while *Trichoderma* is known as a lichen-associated fungus (1). Among the largest contigs were *Nostoc* (89 to 180 kb) (k119_65323, k119_127379, k119_175187, k119_144823, k119_192546, k119_3552, and k119_24448) and mycobiont (87 to 111 kb) (k119_146642, k119_137690, and k119_84554) contigs. The largest *Coccomyxa* contig (k119_27269) was 5 kb in length. The results of the study are of importance to unravel symbiotic interrelationships in lichens.

### Data availability.

The Peltigera canina lichen raw sequencing reads and contigs were deposited in GenBank under BioProject accession number PRJNA756680 and GenBank accession number JAIOUS000000000. The Solorina crocea lichen raw sequencing reads and contigs were deposited in GenBank under BioProject accession number PRJNA756777 and GenBank accession number JAIQCQ000000000. All contigs identified as possible contaminants or adaptors by the NCBI contamination screen were subsequently trimmed or removed during deposition.
